# Effect of Enrofloxacin on the Microbiome, Metabolome, and Abundance of Antibiotic Resistance Genes in the Chicken Cecum

**DOI:** 10.1128/spectrum.04795-22

**Published:** 2023-02-22

**Authors:** Boheng Ma, De Wang, Xueran Mei, Changwei Lei, Cui Li, Hongning Wang

**Affiliations:** a College of Life Sciences, Sichuan University, Chengdu, People’s Republic of China; b Key Laboratory of Bio-Resource and Eco-Environment of the Ministry of Education, Chengdu, People’s Republic of China; c Animal Disease Prevention and Food Safety Key Laboratory of Sichuan Province, Chengdu, People’s Republic of China; d Department of Obstetrics, The Second Clinical Medical College, Jinan University (Shenzhen People’s Hospital), Shenzhen, People’s Republic of China; e Post-doctoral Scientific Research Station of Clinical Medicine, Jinan University, Guangzhou, People’s Republic of China; South China Sea Institute of Oceanology

**Keywords:** gut microbiota, enrofloxacin, metabolome, antibiotic resistance, chicken

## Abstract

Enrofloxacin is an important antibiotic for the treatment of Salmonella infections in livestock and poultry. However, the effects of different concentrations of enrofloxacin on the bacterial and metabolite compositions of the chicken gut and changes in the abundance of resistance genes in cecum contents remain unclear. To investigate the effects of enrofloxacin on chickens, we orally administered different concentrations of enrofloxacin to 1-day-old chickens and performed 16S rRNA gene sequencing to assess changes in the gut microbiomes of chickens after treatment. The abundance of fluoroquinolone (FQ) resistance genes was measured using quantitative PCR. Metabolomics techniques were used to examine the cecal metabolite composition. We found that different concentrations of enrofloxacin had different effects on cecum microorganisms, with the greatest effect on cecum microbial diversity in the low-concentration enrofloxacin group at day 7. Enrofloxacin use reduced the abundance of beneficial bacteria such as *Lactobacillaceae* and *Oscillospira*. Furthermore, cecum microbial diversity was gradually restored as the chickens grew. In addition, enrofloxacin increased the abundance of resistance genes, and there were differences in the changes in abundance among different antibiotic resistance genes. Moreover, enrofloxacin significantly affected linoleic acid metabolism, amino acid metabolism, and signaling pathways. This study helps improve our understanding of how antibiotics affect host physiological activities and provides new insights into the rational use of drugs in poultry farming. The probiotics and metabolites that we identified could be used to modulate the negative effects of antibiotics on the host, which requires further study.

**IMPORTANCE** In this study, we investigated changes in the cecum flora, metabolites, and abundances of fluoroquinolone antibiotic resistance genes in chickens following the use of different concentrations of enrofloxacin. These results were used to determine the effects of enrofloxacin on chick physiology and the important flora and metabolites that might contribute to these effects. In addition, these results could help in assessing the effect of enrofloxacin concentrations on host metabolism. Our findings could help guide the rational use of antibiotics and mitigate the negative effects of antibiotics on the host.

## INTRODUCTION

Because of the overuse of antibiotics in livestock and poultry farming, antibiotic resistance has developed into a major threat to human health (1, 2). Fluoroquinolones (FQs) are a family of synthetic antibacterial drugs, including enrofloxacin, that are widely used for the treatment of Salmonella- and Escherichia coli-related diseases in chickens (3). Due to human health concerns, enrofloxacin use in poultry was banned in the United States in 2005 (4). However, enrofloxacin remains one of the most frequently used antibiotics in poultry farming in China (5–7). Furthermore, it is often administered to newborn chicks to prevent invasion by pathogenic bacteria in modern large-scale intensive poultry farming. FQ-resistant strains have been detected in several farms in different provinces of China (8, 9). Several mechanisms mediating FQ resistance have been identified, among which plasmid-mediated quinolone resistance (PMQR) has been reported to be a key cause of the spread of such resistance (10). The plasmid genes *qnrA*, *qnrB*, *qnrD*, and *qnrS* encode proteins of the pentapeptide repeat sequence family, which protect DNA gyrase and topoisomerase IV from FQ-mediated inhibition (11). FQ resistance can also result from its acetylation with an appropriate amino nitrogen target mediated by a variant of the common aminoglycoside acetyltransferase gene *aac(6′)-Ib-cr* (12). A third mechanism involves the plasmid-based efflux pump-encoding genes *qepA* (13) and *oqxAB* (12, 14), which enhance FQ clearance. In addition, it has been reported that the use of enrofloxacin causes an increase in the abundance of antibiotic resistance genes (ARGs) encoding resistance to macrolide-lincosamide-streptogramin, aminoglycosides, and tetracyclines. *tmexCD1-toprJ1* encodes a resistance-nodulation-division (RND) family efflux pump and is located on a plasmid that confers resistance to quinolones in Gram-negative bacteria (15, 16). Moreover, a change in the abundance of ARGs was found to be correlated with the enrofloxacin concentration (17). However, there is a lack of studies on the effect of different concentrations of enrofloxacin on the abundance of different species with PMQR.

Antibiotics alter the composition and function of the host microbial community (18). Enrofloxacin-exposed zebrafish show significant differences in bacterial communities and intestinal flora richness (19). Changes in taxa with high and low abundances in the pig intestinal flora were also observed after the administration of tylosin (20). Under enrofloxacin stress, the structure of the fecal bacterial community in pigs changes, with a significant increase in the abundance of *Firmicutes*. Furthermore, enrofloxacin-induced differences in the intestinal flora were found to be fully recovered 10 days after the discontinuation of the drug (21). Moreover, corticosterone can affect carbohydrate metabolism and amino acid metabolism in chicks by altering gut microbes (22). In addition, the oral administration of a phage inhibits the cecum lipopolysaccharide biosynthesis pathway in chickens (23). The gut microbiota has also been determined to be associated with the regulation of host immune functions, metabolism, and resistance to pathogenic bacterial colonization (24–26).

Antibiotics also alter the host metabolome. Cefoperazone treatment alters the mouse metabolome, resulting in reduced levels of metabolites, including short-chain fatty acids (SCFAs) (27). SCFA supplementation has been reported to enhance interleukin-22 (IL-22) production, thereby protecting the gut from inflammation (26). Amino acid metabolic pathways and signaling pathways involved in cecum metabolism, which can be affected by antibiotics, have also been shown to be associated with the expansion of pathogenic bacteria such as Salmonella and inflammatory responses (26, 28). Mei et al. reported that florfenicol can affect linoleic acid metabolism in chicks and that linoleic acid supplementation can improve resistance to Salmonella colonization in chicks (29). However, studies are still lacking on the microbiota and metabolite compositions of chicks affected by enrofloxacin, which are important for the rational use of drugs in poultry farming and for a better understanding of the physiological changes in animals after antibiotic intervention. This study could provide a reference for the disruptive effects of enrofloxacin on the intestinal bacterial community and metabolite composition in chickens.

## RESULTS

### Changes in microbial composition and structure after treatment with different concentrations of enrofloxacin.

**FIG 1:**
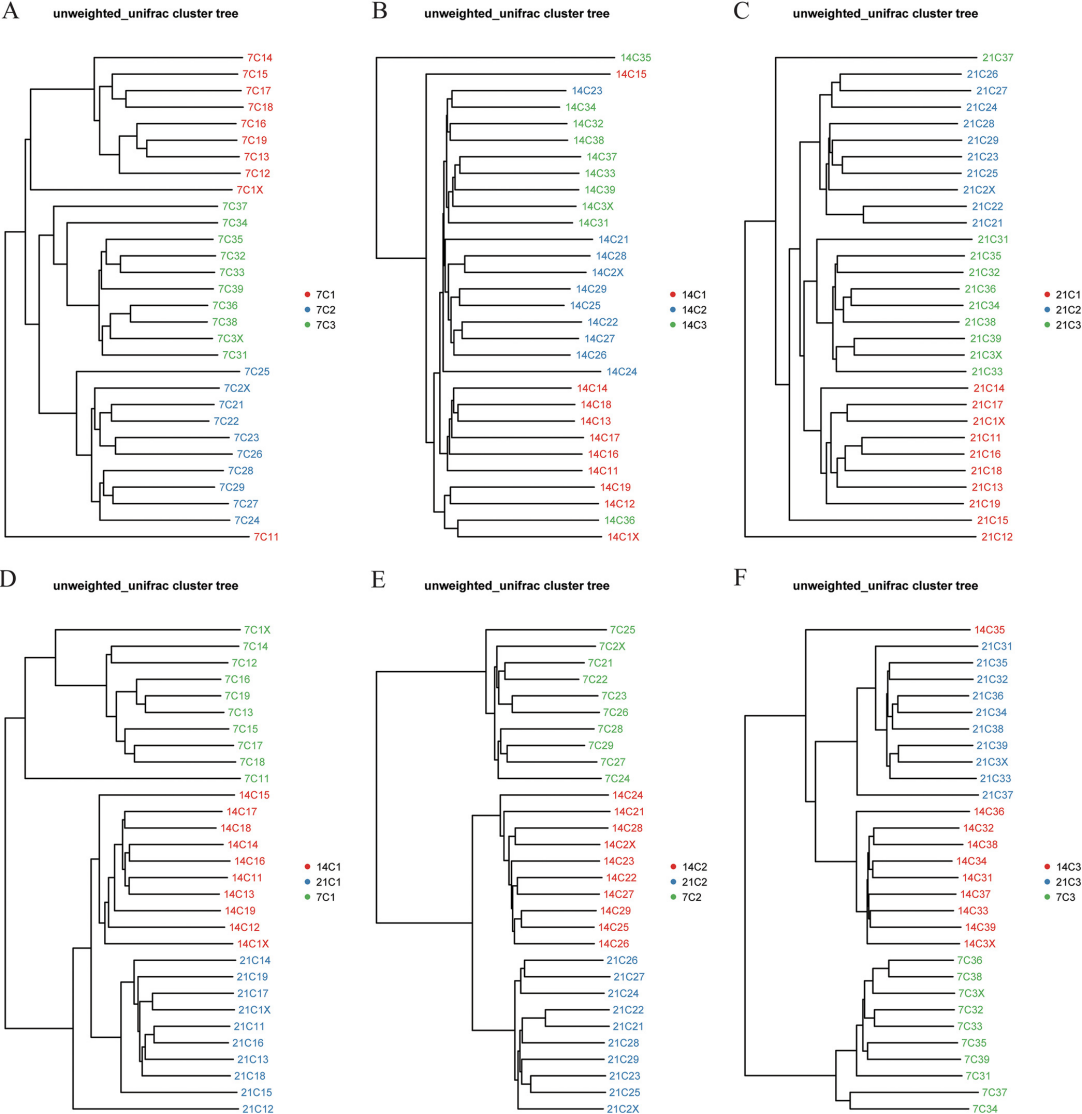
Analysis of the β-diversity of the cecal microbiota after oral administration of different concentrations of enrofloxacin. (A to C) Cluster analysis of samples from the C1, C2, and C3 groups at day 7 (A), day 14 (B), and day 21 (C) to calculate the distances among them. (D to F) Cluster trees of the C1 (D), C2 (E), and C3 (F) groups at the three developmental stages.

**FIG 2:**
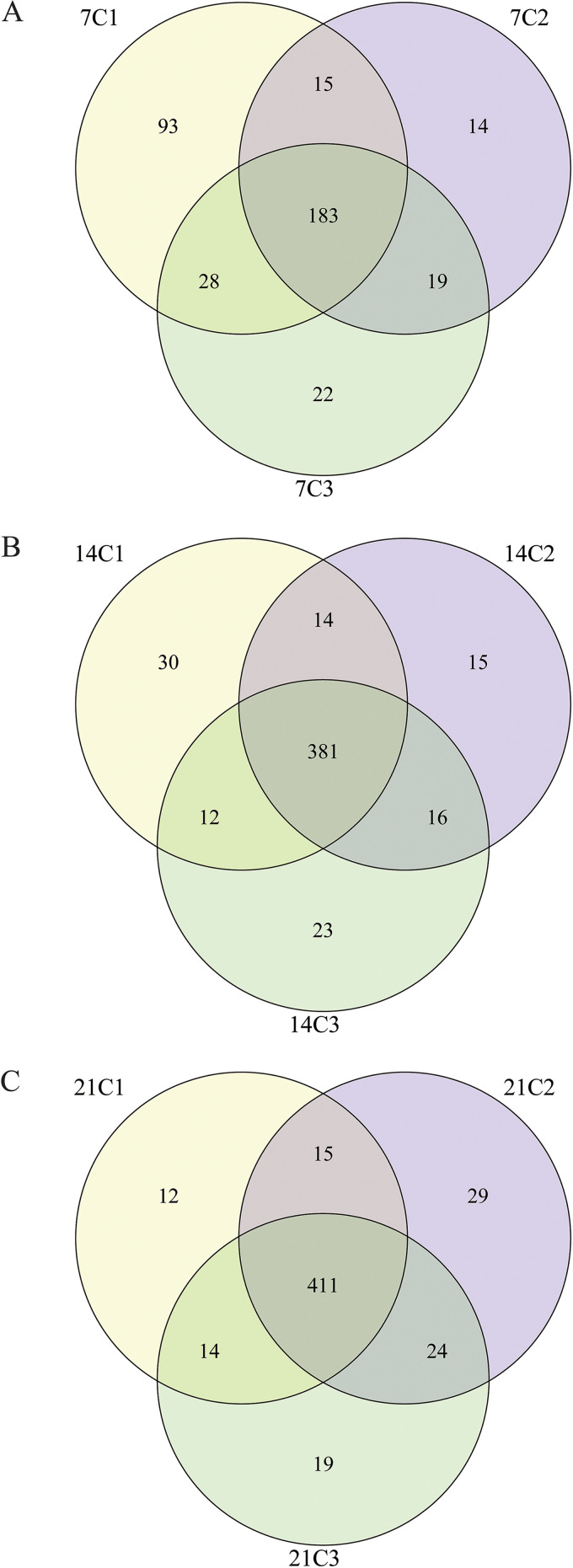
Venn diagram of operational taxonomic units (OTUs) of cecal microorganisms at day 7 (A), day 14 (B), and day 21 (C).

One-day-old chickens were orally administered different concentrations of enrofloxacin for 5 days, as follows: the C1 group was administered 200 μL of distilled water, the C2 group was administered 200 μL of 10 mg/kg of body weight of enrofloxacin, and the C3 group was administered 200 μL of 100 mg/kg of enrofloxacin. A concentration of 10 mg/kg is commonly used in poultry farms to prevent bacterial diseases, whereas 100 mg/kg is the highest nontoxic dose of enrofloxacin for chickens. We sequenced the 16S rRNA genes of the cecum microbiome from chickens at three different developmental stages and used multiple indices to assess the α-diversity of each sample. On day 7, we detected significant differences in microbial diversity among groups C1, C2, and C3 (*P < *0.05). In particular, the untreated C1 group exhibited the highest microbial diversity. The microbial diversity was higher in the C3 group, treated with a high dose of enrofloxacin, than in the C2 group, treated with a low dose. This is consistent with the results of previous studies (19, 30). This may be because high concentrations of enrofloxacin more significantly disrupted the intestinal flora and reduced colonization resistance, favoring an increase in microbial diversity in the early stages of recovery. Interestingly, we did not observe any significant differences in microbial diversity among groups C1, C2, and C3 on days 14 and 21 (see Fig. S1A to C in the supplemental material); all three groups had significantly higher microbial diversity (*P < *0.05) than that at earlier developmental stages (Fig. S1D to F). To analyze the β-diversity of the samples, we used hierarchical clustering to determine the structure of the microbial communities in fecal samples from groups C1, C2, and C3 at the three developmental stages. The different developmental stages were clearly separated in each experimental group (Fig. 1A to C). Likewise, the three experimental groups were also clearly separated at each developmental stage (Fig. 1D to F). We performed a principal-component analysis to analyze the structure of the gut microbiome of each experimental group at the three developmental stages based on absolute operational taxonomic unit (OTU) abundances. We found that the microbiomes of the C1, C2, and C3 groups were different owing to the use of different doses of enrofloxacin. More specifically, at day 7, 183 OTUs were shared among the three experimental groups, with the C1, C2, and C3 groups having 93, 14, and 22 unique OTUs, respectively (Fig. 2A). We observed that at the next two developmental stages, the OTUs shared by the three experimental groups gradually increased. At day 14, 381 OTUs were shared among the three experimental groups, with the C1, C2, and C3 groups having 30, 15, and 23 unique OTUs, respectively, whereas at day 21, 411 OTUs were shared among the three experimental groups, with the C1, C2, and C3 groups having 12, 29, and 19 unique OTUs, respectively (Fig. 2B and C). In addition, partial least-squares discriminant analysis (PLS-DA) revealed significant differences in the structures of the cecal microbial communities among the groups treated with different doses and significant changes in the microbial composition of the cecum in chickens over time (Fig. S2). This result indicates that there are differences in the effects of different concentrations of enrofloxacin on the microorganisms of the cecum in chicks. Although microbial diversity is slowly restored as the chickens develop, differences in microbial community compositions still exist among the different treatment groups.

**FIG 3:**
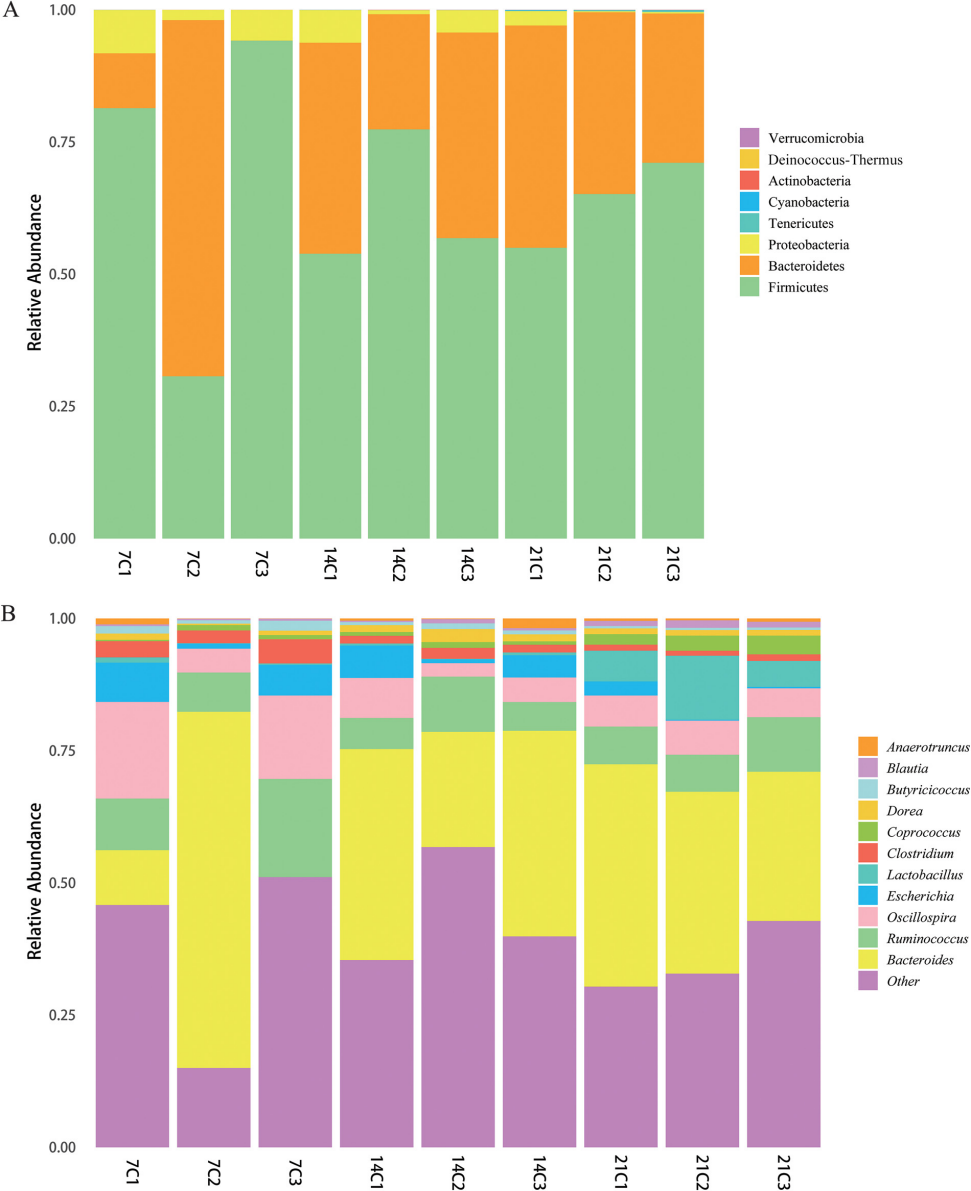
Average relative abundances of microbial species in the cecum at the phylum (A) and genus (B) levels.

We next compared the differences in microbial communities among groups at the phylum (Fig. 3A) and genus (Fig. 3B) levels.The use of different concentrations of enrofloxacin considerably affected the compositions of the cecum microbiomes of the chickens. Notably, *Proteobacteria*, *Bacteroidetes*, and *Firmicutes* were the three most abundant phyla in all experimental groups. However, in the control group (C1), the abundances of *Proteobacteria* and *Firmicutes* gradually decreased over time, whereas the abundance of *Bacteroidetes* gradually increased. In the antibiotic-treated groups (C2 and C3), the abundance of *Firmicutes* was higher whereas that of *Bacteroidetes* was lower than that in the control group. In the C2 group, the abundance of *Firmicutes* increased and then decreased, whereas in group C3, the abundance of *Firmicutes* decreased and then increased. The abundance of *Bacteroidetes* decreased and then increased in group C2. Moreover, in group C3, the abundance of *Bacteroidetes* first increased and then decreased. The abundance of *Proteobacteria* continued to decrease in group C2, whereas in group C3, the abundance of *Proteobacteria* continued to increase. The abundances of the three major groups of *Bacteroidetes* were almost the same among the three groups at day 21 (Table S1). The ratio of *Bacteroidetes* to *Firmicutes* is related to fat accumulation in chickens, and *Bacteroidetes* species have been shown to produce SCFAs to enhance host resistance to pathogenic bacteria. Our results suggest that the use of enrofloxacin might have a negative effect on growth and *Firmicutes* resistance in chicks.

**FIG 4:**
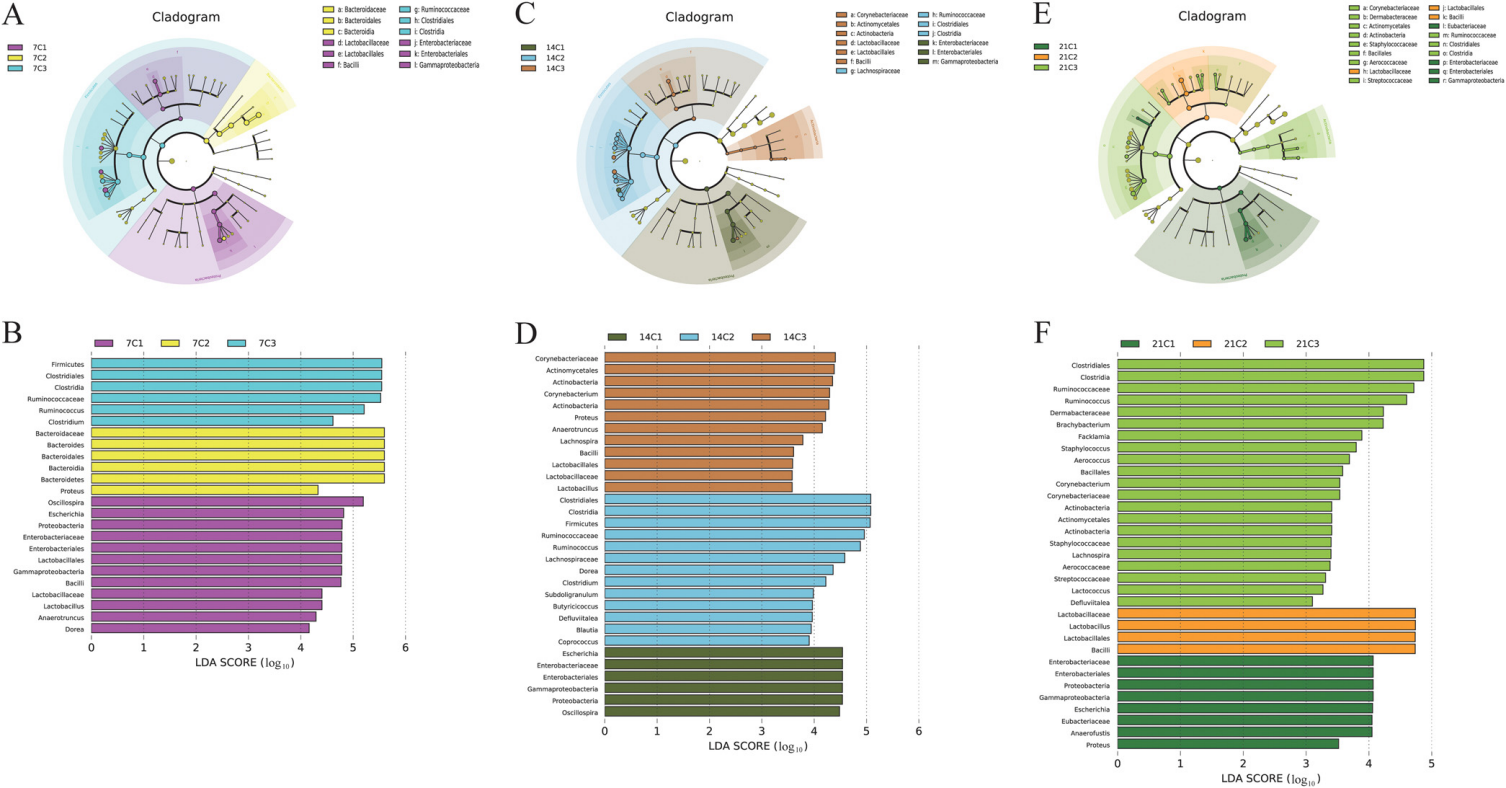
Linear discriminant analysis (LDA) effect size (LEfSe) analysis of chicken cecal microbial communities in groups C1, C2, and C3 at the three developmental stages. (A, C, and E) LEfSe plots showing microbial strains with significant differences among groups C1, C2, and C3. The different groups are represented by different colors. The microbes found to play important roles in the different groups are represented by nodes of the corresponding colors; organism markers are indicated by colored circles. Microbes not found to play significant roles in the different groups are indicated by yellow nodes. From the inside to the outside, circles are ordered according to phylum, class, order, family, and genus. (B, D, and F) LDA diagrams. Different colors represent microbial groups found to play significant roles in groups C1, C2, and C3. Biomarkers associated with significant differences are shown, with histogram colors representing the respective groups and lengths representing LDA scores, which indicate the magnitudes of the effects of significantly different species among groups.

At the genus level (Fig. 3B), enrofloxacin decreased the abundances of *Anaerotruncus*, *Dorea*, *Lactobacillus*, and *Oscillospira* at day 7. The abundances of *Butyricicoccus*, Escherichia, and *Clostridium* gradually decreased over time, whereas the abundances of *Coprococcus* and *Lactobacillus* gradually increased. Multiple cecum microorganisms in the C2 group and the C3 group differed at the genus level among all three developmental stages. This indicates that the concentration of antibiotics affected the structure of the cecum microbial community (Table S2). We also used the linear discriminant analysis (LDA) effect size (LEfSe) method to analyze the effects of different concentrations of enrofloxacin on the intestinal microbiomes of chickens (*P* value of <0.05; LDA score of ≥2). Notably, at day 7, *Lactobacillaceae* and *Oscillospira* comprised the microbial taxa with a significant effect on separating the C1 group from the other two groups (Fig. 4).

### Changes in the metabolomes of cecum contents.

**FIG 5:**
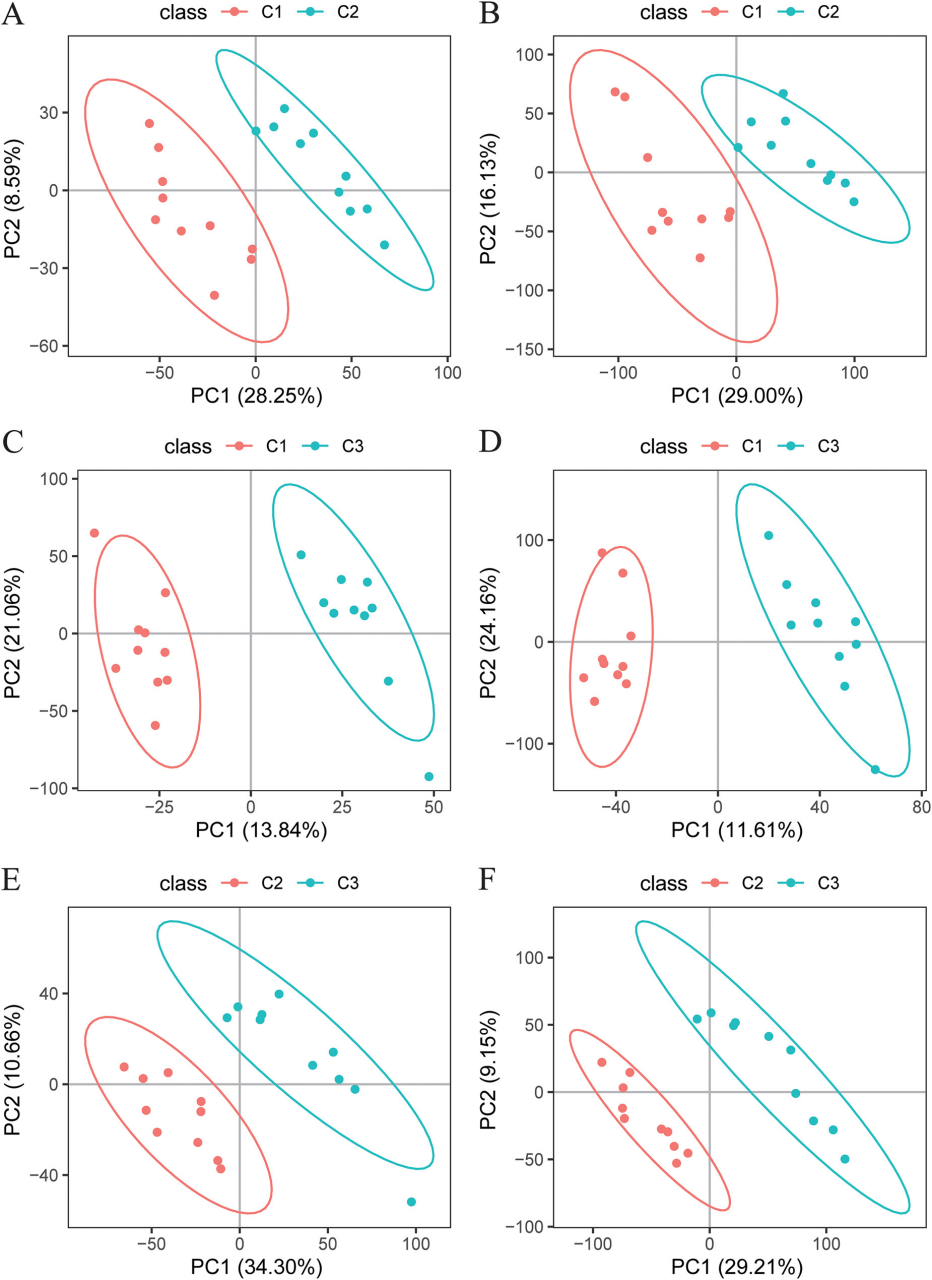
Plot of partial least-squares discriminant analysis (PLS-DA) scores of metabolite compositions in the cecal contents of chickens at day 7. (A) Differential metabolites between the C1 and C2 groups compared in negative-ion mode. (B) Differential metabolites between the C1 and C2 groups compared in positive-ion mode. (C) Differential metabolites between the C1 and C3 groups compared in negative-ion mode. (D) Differential metabolites between the C1 and C3 groups compared in positive-ion mode. (E) Differential metabolites between the C2 and C3 groups compared in negative-ion mode. (F) Differential metabolites in the C2 and C3 groups compared to those in positive-ion mode.

**FIG 6:**
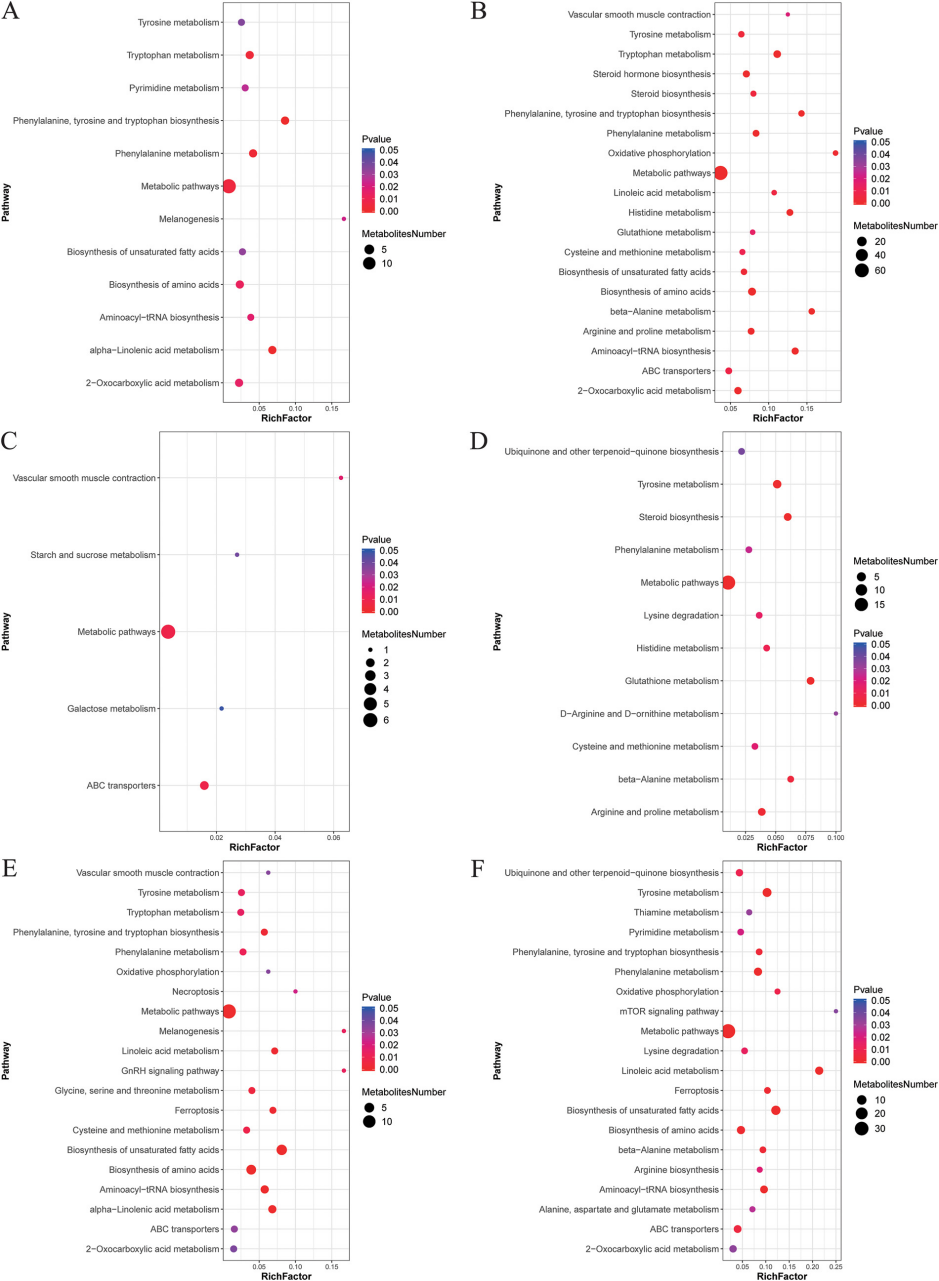
Kyoto Encyclopedia of Genes and Genomes (KEGG) pathway analysis of metabolites in cecal contents at day 7. Metabolites from different comparison groups were analyzed for the degree of enrichment of metabolic pathways according to the KEGG database. The *x* axis (rich factor) indicates the ratio of the number of different metabolites in the pathway to that of all metabolites that were annotated in the pathway. The sizes of the dots represent the numbers of differential metabolites that were annotated to the pathway. (A) Differential metabolites with a negative ion pattern in group C1 versus group C2. (B) Differential metabolites with a positive ion pattern in group C1 versus group C2. (C) Differential metabolites with a negative ion pattern in group C1 versus group C3. (D) Differential metabolites with a positive ion pattern in group C1 versus group C3. (E) Differential metabolites with a negative ion pattern in group C2 versus group C3. GnRH, gonadotropin-releasing hormone. (F) Differential metabolites with a negative ion pattern in group C2 versus group C3 with a positive ion pattern of differential metabolites.

We then investigated the effects of different concentrations of enrofloxacin on the metabolite compositions of chicken cecum contents using untargeted metabolome analysis on day 7. Interestingly, PLS-DA revealed significant differences among the three experimental groups (Fig. 5). The number of significantly differential metabolites among the groups is shown in Table 1 (variable importance in projection [VIP] value of ≥1, fold change of ≥1.2 or ≤0.83, and *q* value of <0.05). Differential metabolites were enriched mainly in amino acid metabolism (e.g., phenylalanine, tyrosine, and tryptophan biosynthesis; arginine and proline metabolism; and histidine metabolism), fatty acid metabolism (e.g., linoleic acid [LA] and α-linolenic acid [ALA] metabolism), sugar metabolism (e.g., galactose, starch, and sucrose metabolism), signaling pathways (e.g., mammalian target of rapamycin [mTOR]), and other metabolic processes (Fig. 6), as determined using Kyoto Encyclopedia of Genes and Genomes (KEGG) pathway enrichment analysis.

**TABLE 1:** Differential metabolites among the groups^*a*^

Group comparison	Positive-ion mode		Negative-ion mode	
	No. of significantly different metabolites	Trend (no. increased, no. decreased)	No. of significantly different metabolites	Trend (no. increased, no. decreased)
C2 vs C1	2,818	1,474↑, 1,344↓	1,079	603↑, 476↓
C3 vs C1	545	224↑, 321↓	263	116↑, 147↓
C3 vs C2	1,594	776↑, 818↓	618	357↑, 261↓

a Differential metabolite species were considered those with a VIP value of ≥1, a fold change of ≥1.2 or ≤0.83, and a *q* value of <0.05.

### Variations in the abundance of antibiotic resistance genes.

**FIG 7:**
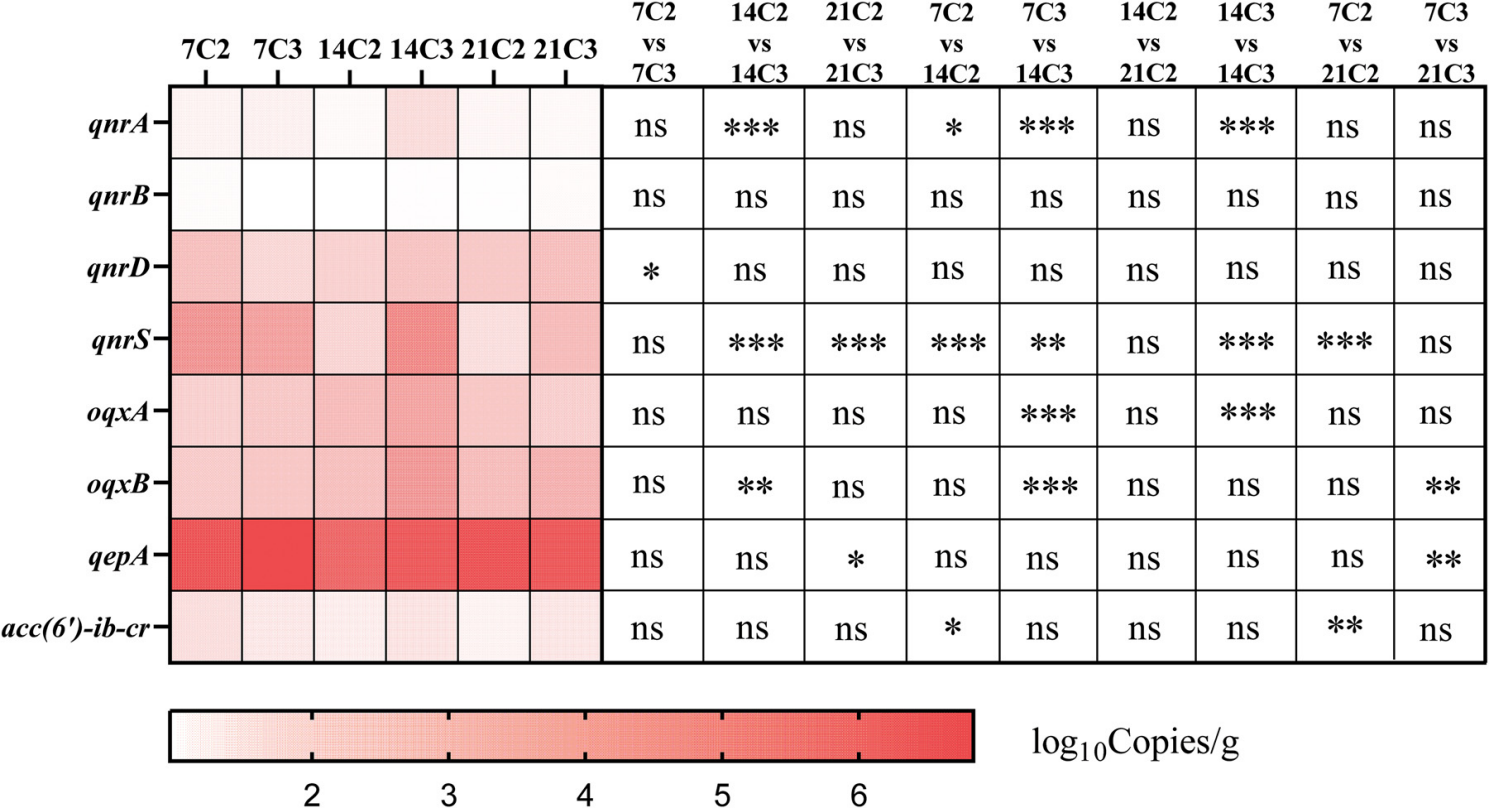
Heat map of relative antibiotic resistance gene (ARG) abundances for each group. The *x* axis represents different subgroups, whereas the *y* axis represents different genes. The right side shows the differences in ARG abundances among the different groups. The copy number of ARGs was converted to log_10_ copies per gram. ARG abundances were analyzed using one-way analysis of variance with SPSS 20.0 software. ***, *P < *0.001; **, 0.001 ≤ *P ≤ *0.01; *, 0.01 ≤ *P ≤ *0.05; ns, not significant (*P > *0.05).

We finally assessed the abundance of PMQR genes in cecum contents using quantitative PCR (qPCR). We did not detect any relevant genes in the C1 group. Conversely, in the C2 and C3 groups, the abundances of *qnrD*, *qnrS*, *oqxA*, *oqxB*, and *qepA* were higher than those of the other resistance genes (Fig. 7). This may be caused by the increased abundance of resistance genes in the C2 and C3 groups under the selection pressure of enrofloxacin, while the abundance of resistance genes in the C1 group was below the detection limit. Among the genes, *qnrA* and *qnrB* were the least abundant, whereas *qepA* showed the highest abundance in all groups and at all three developmental stages. Moreover, the abundances of the *qnrA*, *qnrS*, and *oqxB* genes were significantly different among the groups (*P < *0.05), suggesting that the abundance of these resistance genes might be influenced by antibiotic concentration and time. This is consistent with previously reported results showing that antibiotic concentration and time can significantly affect the abundance of resistance genes (31–33).

## DISCUSSION

Enrofloxacin is currently the antibiotic of choice for the prevention and treatment of Salmonella infections in livestock and poultry. However, it causes antibiotic resistance problems, and disturbances to the host cecum flora and metabolism have been identified as being potential pathogenic mechanisms leading to disease pathogenesis (34). Here, we investigated the effects of enrofloxacin on the cecum flora and metabolites and PMQR gene abundances in chickens by feeding them different concentrations of enrofloxacin. Our results showed that α-diversity was highest in the C1 group at day 7, followed by the C3 group, and lowest in the C2 group. There was no significant difference in α-diversity among the three groups at days 14 and 21 (*P > *0.05). This is consistent with previously reported results indicating that the diversity of the cecum microbiota is disrupted after the use of antibiotics (17, 35, 36). Moreover, with age, the cecum microbiota expands gradually. Higher microbial diversity tends to indicate that the host has greater disease resistance. Previous studies have shown that piglets with higher α-diversity have lower inflammatory factor levels and a greater antioxidant capacity in the intestine, and the protein expression levels of ZO-1, occludin, and claudin-1 are also higher (37). Furthermore, α-diversity is associated with feed efficiency (38). In a study of Canadian patients with Crohn’s disease, α-diversity was lower in those with the disease than in the healthy population (39). Therefore, the use of enrofloxacin might result in reduced feed efficiency and increased susceptibility to disease in chickens.

Our results showed that the use of enrofloxacin significantly alters the structure of the cecum community. Although there was no significant difference in α-diversity values among the three groups at days 14 and 21, it was evident from the PLS-DA that different concentrations of enrofloxacin profoundly altered the cecum microbial structure. Similar to the results of previous studies, the chick gut microbial community is dominated by *Firmicutes*, *Bacteroidetes*, and *Proteobacteria* (40). Notably, the ratio of *Bacteroidetes* to *Firmicutes* was higher in the C2 group than in the other groups at day 7. Previous studies have reported that an increased ratio of *Bacteroidetes* to *Firmicutes* results in a decrease in the percentage of abdominal fat in chickens. This suggests that enrofloxacin might affect the growth of chickens (41). Furthermore, the abundance of *Bacteroidetes* in the C3 group was significantly lower than that in the C1 group at day 7. It has been reported that *Bacteroidetes* species can restore host immunity and enhance pathogen clearance by producing butyrate, thereby reversing the process of sepsis (42). This could be one of the reasons why chicks are more susceptible to pathogenic bacteria after being administered antibiotics. Our results indicated that *Lactobacillaceae* and *Oscillospira* abundances were significantly different between the C2 and C3 groups and the C1 group at the genus level on day 7. Bacteriocins produced by *Lactobacillaceae* can directly inhibit the invasion of competing strains or pathogens or modulate the composition of the microbiota and affect the host immune system (43). The *Oscillospira* abundance is significantly reduced in patients with Crohn’s disease (44), and it might be capable of producing SCFAs (45). SCFAs serve as an important fuel for intestinal epithelial cells (IECs), and they can regulate IEC proliferation and differentiation, influence intestinal motility, and strengthen intestinal barrier function and host metabolism (46, 47). Therefore, our results suggest that enrofloxacin decreases the diversity of the intestinal flora and reduces the abundance of beneficial intestinal bacteria, which in turn might affect host resistance and growth performance in response to pathogenic bacteria.

We chose the cecum contents at day 7 for metabolomic determinations because the differences in microbial compositions among the groups were greatest at this time. Our results showed a significant difference in the compositions of cecum contents among the groups at day 7. The differential metabolic pathways between the C1 and C2 groups included α-linolenic acid (ALA) metabolism and linoleic acid (LA) metabolism. Both ALA and LA are omega-3 polyunsaturated fatty acids (PUFAs). According to previous studies, the administration of an ALA-rich diet reduces interleukin-1β (IL-1β), tumor necrosis factor alpha, IL-6, and IL-17A production in mice, along with increased SCFA production (48). Supplementing the diets of pigeons with LA improves their antioxidant capacity and enhances their lipolytic capacity (49). According to a previous meta-analysis, ALA improves lipid profiles by lowering triglyceride, total cholesterol, high-density lipoprotein (HDL) cholesterol, low-density lipoprotein (LDL), and very-low-density lipoprotein levels (50). The oral administration of omega-3 PUFAs reduces inflammation in obese mice, thereby enhancing the function of the immune system (51). Enrofloxacin use alters the mTOR signaling pathway. mTOR is a highly conserved serine/threonine protein kinase that consists of two forms of the complex, and the activation of mTOR is associated with a complex set of signaling pathways, including inflammatory responses (52). After colitis is induced by lipopolysaccharide in mice, the upstream Toll-like receptor 4 (TLR4) signaling pathway activates mTOR and inhibits autophagy. mTOR-dependent autophagy coordinates downstream NF-κB activation, leading to proinflammatory cytokine production and oxidative stress (53). In summary, the use of enrofloxacin might affect the inflammatory response of chicks by altering unsaturated fatty acid metabolism and the mTOR signaling pathway in a manner that disrupts normal intestinal function in chickens.

The results of the antibiotic resistance gene abundance analysis showed an increase in the abundance of PMQR genes in the cecum contents after enrofloxacin administration. However, different patterns of changes in the abundances of different genes were observed. This is similar to the results of previous studies. Zhang et al. previously reported that the effect of chlortetracycline on a fecal resistance group depends on the specific ARG subtypes rather than the overall ARG levels. The change in the structure of the fecal microbial community induced by the antibiotic was found to be accompanied by a change in the abundance of bacterial hosts carrying a specific ARG (54). We found no significant changes in partial PMQR gene abundances at day 14 compared to those at days 7 and 21 (*P > *0.05). This is similar to previous reports showing that the abundance of antibiotic resistance genes is not changed significantly after a period of antibiotic discontinuation (55). In addition, according to previous reports, the use of antibiotics might cause changes in the abundance of genes conferring resistance to other types of antibiotics (56). The pattern of the change in the abundance of genes conferring resistance to other types of antibiotics after the use of enrofloxacin needs further study. The risk of transmission of ARGs from animals to humans should also be comprehensively assessed.

In conclusion, our results describe changes in the cecum microbiome, metabolite composition, and abundance of resistance genes in chicks following enrofloxacin use. These changes might affect the growth and development of chicks as well as their resistance to pathogenic bacterial invasion. The differential metabolites screened could provide important insights to mitigate the effects of antibiotics. Moreover, the results of this study could facilitate the more prudent and rational use of antibiotics in the poultry industry.

## MATERIALS AND METHODS

### Infection of chickens and exposure to enrofloxacin.

The animal experimental protocol was approved by the Animal Ethics Committee of Sichuan University (Chengdu, Sichuan Province, People’s Republic of China). Ninety 1-day-old Lohmann chickens free of specific pathogens were randomly divided into three groups with three replicates each. For each replicate, 10 chickens were kept in the same cage. These chickens had an average weight of approximately 50 g and were kept in cages under standard commercial conditions. Each chicken was given free access to standard antibiotic-free commercial corn-soybean feed (Yellow little chicken feed; Guangdong Wens Dahuanong Biotechnology Co., Ltd.) and water. Subsequently, 1-day-old chickens were orally administered water or enrofloxacin (Baytril 10% injectable solution; Bayer AG, Leverkusen, Germany) for 5 consecutive days (group C1, 200 μL distilled water; group C2, 200 μL 10 mg/kg enrofloxacin [standard therapeutic concentration {57}]; group C3, 200 μL 100 mg/kg enrofloxacin [maximum nontoxic concentration {58}]). Chickens were euthanized using a 5% pentobarbital solution (50 mg/kg of body weight) on days 7, 14, and 21, and their fecal contents were collected (59).

### DNA extraction.

For DNA extraction, 200 mg of cecal contents was collected from 10 chickens from each group at the above-mentioned sampling time points. Total genomic DNA was extracted using the Qiagen DNA mini stool kit (Qiagen, Hilden, Germany) according to the manufacturer’s instructions. The concentration and quality of isolated DNA were assessed using a NanoDrop spectrophotometer (Thermo Fisher Scientific, Waltham, MA, USA) and agarose gel electrophoresis. The obtained DNA was stored at −20°C pending further analysis.

### 16S rRNA amplicon sequencing.

The V3 and V4 variable regions of the bacterial 16S rRNA gene were amplified with degenerate PCR primers, specifically primers 341F (5′-ACTCCTACGGGAGGCAGCAG-3′) and 806R (5′-GGACTACHVGGGTWTCTAAT-3′). PCR enrichment was performed in a 50-μL reaction mixture containing 30 ng of the template, a fusion PCR primer, and PCR master mix. PCR cycling conditions were as follows: 94°C for 3 min; 30 cycles of 94°C for 30 s, 56°C for 45 s, and 72°C for 45 s; and a final extension step at 72°C for 10 min. PCR products were purified using AMPure XP magnetic beads. The Agilent 2100 Bioanalyzer (Agilent Technologies, Santa Clara, CA, USA) was used to validate the library fragment range and concentration, and validated libraries were sequenced on an Illumina HiSeq platform.

### Analysis of 16S rRNA sequencing data.

Raw reads were filtered to remove adapters and low-quality bases to obtain clean data. Sequence splicing was performed using FLASH v1.2.11 software (60), which assembled paired reads from paired-end sequencing into sequences using overlapping relationships. Using USEARCH v7.0.1090 software (61), the spliced tags were clustered into OTUs at 97% similarity. The representative OTU sequences were classified and analyzed using RDP classifier v2.2 (62). USEARCH_global was used to compare all tags back to OTUs to obtain the OTU abundance statistics table for each sample (63).

### Bioinformatics and statistical analyses.

α- and β-diversity values were estimated using MOTHUR (v1.31.2) (64) and QIIME (v1.8.0) (65), respectively, at the OTU level. LEfSe cluster analysis or LDA was conducted using the LEfSe algorithm. PLS-DA was performed using the R package mixOmics. Bar plots of different classification levels were plotted using the R package v3.4.1 and the gplots R package. Significant species or functions were determined using R (v3.4.1) based on a Wilcoxon test or a Kruskal-Wallis test.

### Untargeted metabolomic analysis of chicken cecal contents.

The cecal contents at day 7 were slowly thawed at 4°C and weighed to 25 mg in a 1.5-mL tube. Subsequently, 800 μL of extraction solution (methanol-acetonitrile-water at 2:2:1 [vol/vol/vol], prechilled at −20°C), 10 μL of internal standard 1, and 10 μL of internal standard 2 were added to each sample along with two small steel balls; next, each sample was placed into a tissue grinder for grinding (50 Hz for 5 min), sonicated in a water bath at 4°C for 10 min, and placed at −20°C for 1 h. After centrifugation at 25,000 × *g* for 15 min at 4°C, 600 μL of the supernatant was placed into a cryo-vacuum concentrator and resuspended in 200 μL of reagent solution (methanol-H_2_O at 1:9 [vol/vol]) with vortexing. The supernatant was centrifuged at 25,000 × *g* for 15 min after sonication in a water bath at 4°C for 10 min and placed into a sample bottle. An Acquity two-dimensional (2D) ultraperformance liquid chromatograph system (Waters, USA) with a Q Exactive Plus high-resolution mass spectrometer (Thermo Fisher Scientific) was used for the separation and detection of metabolites.

The raw data acquired via liquid chromatography-tandem mass spectrometry (LC-MS/MS) were imported into Compound Discoverer 3.0 software (Thermo Fisher Scientific) for data processing. The results exported from Compound Discoverer 3.0 were imported into metaX for data preprocessing (66). A combination of multivariate statistical analysis (PLS-DA) (67) and univariate analysis (fold change and Student’s *t* tests) was used to screen for between-group differential metabolites. Data were analyzed using clustering analysis, log_2_ conversion, and z-score normalization (zero-mean normalization). Hierarchical clustering was used in the clustering algorithm, and the Euclidean distance was used to calculate the metabolic pathway enrichment of differential metabolites in the KEGG database. Metabolic pathway enrichment analysis was performed for differential metabolites based on the KEGG database. Metabolic pathways with *P* values of <0.05 were those that were significantly enriched based on differential metabolites.

### Validation of changes in the abundance of cecal antibiotic resistance genes using qPCR.

Total genomic DNA was extracted as described above. qPCR was performed using a Bio-Rad real-time PCR detection system (CFX Maestro 1.1 or 3.0; Bio-Rad Inc., Hercules, CA, USA) and SsoFast EvaGreen supermix (Bio-Rad Inc.) according to the manufacturer’s instructions. Primer sequences for target genes are shown in Table S3 in the supplemental material. The reaction protocol for all genes was as follows: 5 min at 95°C followed by 40 cycles of 10 s at 95°C and 30 s at 60°C. A dilution gradient of a pMD19-T plasmid (TaKaRa, Dalian, China) containing the target genes was used to obtain a standard curve, according to which the absolute expression levels of target genes were calculated.

### Statistical analysis.

The copy numbers of ARGs were converted to log_10_ copies per gram. ARG abundance was analyzed using one-way analysis of variance with SPSS 20.0 software (IBM, Armonk, NY, USA).

### Data availability.

The 16S rRNA amplicon sequencing data from this study were submitted to the NCBI under BioProject accession no. PRJNA647992. The raw data for the metabolome have been uploaded to Metabolights under identifier MTBLS6912 (https://www.ebi.ac.uk/metabolights/MTBLS6912). It is anticipated that this accession number will be released by March 15, 2023. Until then, data will be available from the corresponding author (68).

## References

[1] Raboisson D, Ferchiou A, Sans P, Lhermie G, Dervillé M. 2020. The economics of antimicrobial resistance in veterinary medicine: optimizing societal benefits through mesoeconomic approaches from public and private perspectives. One Health 10:100145. doi:10.1016/j.onehlt.2020.100145. 33117866 PMC7582218

[2] Vekemans J, Hasso-Agopsowicz M, Kang G, Hausdorff WP, Fiore A, Tayler E, Klemm EJ, Laxminarayan R, Srikantiah P, Friede M, Lipsitch M. 2021. Leveraging vaccines to reduce antibiotic use and prevent antimicrobial resistance: a World Health Organization action framework. Clin Infect Dis 73:e1011–e1017. doi:10.1093/cid/ciab062. 33493317 PMC8366823

[3] Gouvêa R, Dos Santos F, De Aquino M. 2015. Fluoroquinolones in industrial poultry production, bacterial resistance and food residues: a review. Braz J Poult Sci 17:1–10. doi:10.1590/1516-635x17011-10.

[4] Center for Veterinary Medicine, US Food and Drug Administration. 2005. Withdrawal of enrofloxacin for poultry. US Food and Drug Administration, Silver Spring, MD. https://www.fda.gov/animal-veterinary/recalls-withdrawals/withdrawal-enrofloxacin-poultry.

[5] Ma X, Chen L, Yin L, Li Y, Yang X, Yang Z, Li G, Shan H. 2022. Risk analysis of 24 residual antibiotics in poultry eggs in Shandong, China (2018–2020). Vet Sci 9:126. doi:10.3390/vetsci9030126. 35324854 PMC8953159

[6] Kang J, Hossain MA, Park H-C, Jeong OM, Park S-W, Her M. 2021. Cross-contamination of enrofloxacin in veterinary medicinal and nutritional products in Korea. Antibiotics (Basel) 10:128. doi:10.3390/antibiotics10020128. 33572763 PMC7912672

[7] Teglia CM, Guiñez M, Culzoni MJ, Cerutti S. 2021. Determination of residual enrofloxacin in eggs due to long term administration to laying hens. Analysis of the consumer exposure assessment to egg derivatives. Food Chem 351:129279. doi:10.1016/j.foodchem.2021.129279. 33631615

[8] Chen Z, Bai J, Zhang X, Wang S, Chen K, Lin Q, Xu C, Qu X, Zhang H, Liao M, Zhang J. 2021. Highly prevalent multidrug resistance and QRDR mutations in Salmonella isolated from chicken, pork and duck meat in southern China, 2018–2019. Int J Food Microbiol 340:109055. doi:10.1016/j.ijfoodmicro.2021.109055. 33485100

[9] Tang B, Elbediwi M, Nambiar RB, Yang H, Lin J, Yue M. 2022. Genomic characterization of antimicrobial-resistant Salmonella enterica in duck, chicken, and pig farms and retail markets in eastern China. Microbiol Spectr 10:e01257-22. doi:10.1128/spectrum.01257-22. PMC9603869 36047803

[10] Li J, Hao H, Dai M, Zhang H, Ning J, Cheng G, Shabbir MAB, Sajid A, Yuan Z. 2019. Resistance and virulence mechanisms of Escherichia coli selected by enrofloxacin in chicken. Antimicrob Agents Chemother 63:e01824-18. doi:10.1128/AAC.01824-18. 30803968 PMC6496110

[11] Ruiz J. 2019. Transferable mechanisms of quinolone resistance from 1998 onward. Clin Microbiol Rev 32:e00007-19. doi:10.1128/CMR.00007-19. 31413045 PMC6730498

[12] Chan BK-W, Wong MH-Y, Chan EW-C, Chen S. 2022. Transcriptional regulation and functional characterization of the plasmid-borne oqxAB genes in Salmonella Typhimurium. Microbiol Spectr 10:e02170-21. doi:10.1128/spectrum.02170-21. 35315694 PMC9045139

[13] Cheng P, Yang Y, Li F, Li X, Liu H, Fazilani SA, Guo W, Xu G, Zhang X. 2020. The prevalence and mechanism of fluoroquinolone resistance in Escherichia coli isolated from swine farms in China. BMC Vet Res 16:258. doi:10.1186/s12917-020-02483-4. 32723358 PMC7388466

[14] Li J, Zhang H, Ning J, Sajid A, Cheng G, Yuan Z, Hao H. 2019. The nature and epidemiology of OqxAB, a multidrug efflux pump. Antimicrob Resist Infect Control 8:44. doi:10.1186/s13756-019-0489-3. 30834112 PMC6387526

[15] Lv L, Wan M, Wang C, Gao X, Yang Q, Partridge SR, Wang Y, Zong Z, Doi Y, Shen J, Jia P, Song Q, Zhang Q, Yang J, Huang X, Wang M, Liu JH. 2020. Emergence of a plasmid-encoded resistance-nodulation-division efflux pump conferring resistance to multiple drugs, including tigecycline, in Klebsiella pneumoniae. mBio 11:e02930-19. doi:10.1128/mBio.02930-19. 32127452 PMC7064769

[16] Dong N, Zeng Y, Wang Y, Liu C, Lu J, Cai C, Liu X, Chen Y, Wu Y, Fang Y, Fu Y, Hu Y, Zhou H, Cai J, Hu F, Wang S, Wang Y, Wu Y, Chen G, Shen Z, Chen S, Zhang R. 2022. Distribution and spread of the mobilised RND efflux pump gene cluster tmexCD-toprJ in clinical Gram-negative bacteria: a molecular epidemiological study. Lancet Microbe 3:e846–e856. doi:10.1016/S2666-5247(22)00221-X. 36202114

[17] Temmerman R, Ghanbari M, Antonissen G, Schatzmayr G, Duchateau L, Haesebrouck F, Garmyn A, Devreese M. 2022. Dose-dependent impact of enrofloxacin on broiler chicken gut resistome is mitigated by synbiotic application. Front Microbiol 13:869538. doi:10.3389/fmicb.2022.869538. 35992659 PMC9386515

[18] Becattini S, Taur Y, Pamer EG. 2016. Antibiotic-induced changes in the intestinal microbiota and disease. Trends Mol Med 22:458–478. doi:10.1016/j.molmed.2016.04.003. 27178527 PMC4885777

[19] Qiu W, Liu T, Liu X, Chen H, Luo S, Chen Q, Magnuson JT, Zheng C, Xu EG, Schlenk D. 2022. Enrofloxacin induces intestinal microbiota-mediated immunosuppression in zebrafish. Environ Sci Technol 56:8428–8437. doi:10.1021/acs.est.1c08712. 35545936 PMC9228068

[20] Yan H, Yu B, Degroote J, Spranghers T, Van Noten N, Majdeddin M, Van Poucke M, Peelman L, De Vrieze J, Boon N, Gielen I, Smet S, Chen D, Michiels J. 2020. Antibiotic affects the gut microbiota composition and expression of genes related to lipid metabolism and myofiber types in skeletal muscle of piglets. BMC Vet Res 16:392. doi:10.1186/s12917-020-02592-0. 33066774 PMC7568366

[21] Chen T, Xie G, Mi J, Wen X, Cao Z, Ma B, Zou Y, Zhang N, Wang Y, Liao X, Wu Y. 2022. Recovery of the structure and function of the pig manure bacterial community after enrofloxacin exposure. Microbiol Spectr 10:e02004-21. doi:10.1128/spectrum.02004-21. 35604139 PMC9241743

[22] Zaytsoff SJM, Uwiera RRE, Inglis GD. 2020. Physiological stress mediated by corticosterone administration alters intestinal bacterial communities and increases the relative abundance of Clostridium perfringens in the small intestine of chickens. Microorganisms 8:1518. doi:10.3390/microorganisms8101518. 33019786 PMC7650536

[23] Zhao H, Li Y, Lv P, Huang J, Tai R, Jin X, Wang J, Wang X. 2022. Salmonella phages affect the intestinal barrier in chicks by altering the composition of early intestinal flora: association with time of phage use. Front Microbiol 13:947640. doi:10.3389/fmicb.2022.947640. 35910610 PMC9329052

[24] Hooper LV, Littman DR, Macpherson AJ. 2012. Interactions between the microbiota and the immune system. Science 336:1268–1273. doi:10.1126/science.1223490. 22674334 PMC4420145

[25] Rogers AWL, Tsolis RM, Bäumler AJ. 2021. Salmonella versus the microbiome. Microbiol Mol Biol Rev 85:e00027-19. doi:10.1128/MMBR.00027-19. 33361269 PMC8549850

[26] Yang W, Yu T, Huang X, Bilotta AJ, Xu L, Lu Y, Sun J, Pan F, Zhou J, Zhang W, Yao S, Maynard CL, Singh N, Dann SM, Liu Z, Cong Y. 2020. Intestinal microbiota-derived short-chain fatty acids regulation of immune cell IL-22 production and gut immunity. Nat Commun 11:4457. doi:10.1038/s41467-020-18262-6. 32901017 PMC7478978

[27] Theriot CM, Koenigsknecht MJ, Carlson PE, Jr, Hatton GE, Nelson AM, Li B, Huffnagle GB, Li JZ, Young VB. 2014. Antibiotic-induced shifts in the mouse gut microbiome and metabolome increase susceptibility to Clostridium difficile infection. Nat Commun 5:3114. doi:10.1038/ncomms4114. 24445449 PMC3950275

[28] Mohseni AH, Casolaro V, Bermúdez-Humarán LG, Keyvani H, Taghinezhad-S S. 2021. Modulation of the PI3K/Akt/mTOR signaling pathway by probiotics as a fruitful target for orchestrating the immune response. Gut Microbes 13:1886844. doi:10.1080/19490976.2021.1886844. 33615993 PMC7899637

[29] Mei X, Ma B, Zhai X, Zhang A, Lei C, Zuo L, Yang X, Zhou C, Wang H. 2021. Florfenicol enhances colonization of a Salmonella enterica serovar Enteritidis floR mutant with major alterations to the intestinal microbiota and metabolome in neonatal chickens. Appl Environ Microbiol 87:e01681-21. doi:10.1128/AEM.01681-21. 34613752 PMC8612288

[30] Keijser BJF, Agamennone V, van den Broek TJ, Caspers M, van de Braak A, Bomers R, Havekes M, Schoen E, van Baak M, Mioch D, Bomers L, Montijn RC. 2019. Dose-dependent impact of oxytetracycline on the veal calf microbiome and resistome. BMC Genomics 20:65. doi:10.1186/s12864-018-5419-x. 30660184 PMC6339435

[31] Tu Z, Shui J, Liu J, Tuo H, Zhang H, Lin C, Feng J, Feng Y, Su W, Zhang A. 2023. Exploring the abundance and influencing factors of antimicrobial resistance genes in manure plasmidome from swine farms. J Environ Sci (China) 124:462–471. doi:10.1016/j.jes.2021.11.030. 36182154

[32] Kayani MUR, Yu K, Qiu Y, Yu X, Chen L, Huang L. 2022. Longitudinal analysis of exposure to a low concentration of oxytetracycline on the zebrafish gut microbiome. Front Microbiol 13:985065. doi:10.3389/fmicb.2022.985065. 36212820 PMC9536460

[33] Sun W, Qian X, Wang X, Gu J. 2023. Residual enrofloxacin in cattle manure increased persistence and dissemination risk of antibiotic resistance genes during anaerobic digestion. J Environ Manage 326:116864. doi:10.1016/j.jenvman.2022.116864. 36436244

[34] Fenneman AC, Weidner M, Chen LA, Nieuwdorp M, Blaser MJ. 2023. Antibiotics in the pathogenesis of diabetes and inflammatory diseases of the gastrointestinal tract. Nat Rev Gastroenterol Hepatol 20:81–100. doi:10.1038/s41575-022-00685-9. 36258032 PMC9898198

[35] Kairmi SH, Taha-Abdelaziz K, Yitbarek A, Sargolzaei M, Spahany H, Astill J, Shojadoost B, Alizadeh M, Kulkarni RR, Parkinson J, Sharif S. 2022. Effects of therapeutic levels of dietary antibiotics on the cecal microbiome composition of broiler chickens. Poult Sci 101:101864. doi:10.1016/j.psj.2022.101864. 35477134 PMC9061639

[36] Zhang N, Liu J, Chen Z, Chen N, Gu F, He Q. 2022. Integrated analysis of the alterations in gut microbiota and metabolites of mice induced after long-term intervention with different antibiotics. Front Microbiol 13:832915. doi:10.3389/fmicb.2022.832915. 35847062 PMC9277126

[37] Cui C, Wu C, Wang J, Ma Z, Zheng X, Zhu P, Wang N, Zhu Y, Guan W, Chen F. 2022. Restored intestinal integrity, nutrients transporters, energy metabolism, antioxidative capacity and decreased harmful microbiota were associated with IUGR piglet’s catch-up growth before weanling. J Anim Sci Biotechnol 13:129. doi:10.1186/s40104-022-00770-8. 36229888 PMC9564052

[38] Bergamaschi M, Tiezzi F, Howard J, Huang YJ, Gray KA, Schillebeeckx C, McNulty NP, Maltecca C. 2020. Gut microbiome composition differences among breeds impact feed efficiency in swine. Microbiome 8:110. doi:10.1186/s40168-020-00888-9. 32698902 PMC7376719

[39] Leibovitzh H, Lee S-H, Xue M, Raygoza Garay JA, Hernandez-Rocha C, Madsen KL, Meddings JB, Guttman DS, Espin-Garcia O, Smith MI, Goethel A, Griffiths AM, Moayyedi P, Steinhart AH, Panaccione R, Huynh HQ, Jacobson K, Aumais G, Mack DR, Abreu MT, Bernstein CN, Marshall JK, Turner D, Xu W, CCC GEM Project Research Consortium, Turpin W, Croitoru K. 2022. Altered gut microbiome composition and function are associated with gut barrier dysfunction in healthy relatives of patients with Crohn’s disease. Gastroenterology 163:1364–1376.e10. doi:10.1053/j.gastro.2022.07.004. 35850197

[40] Khan S, Moore RJ, Stanley D, Chousalkar KK. 2020. The gut microbiota of laying hens and its manipulation with prebiotics and probiotics to enhance gut health and food safety. Appl Environ Microbiol 86:e00600-20. doi:10.1128/AEM.00600-20. 32332137 PMC7301851

[41] Xiang H, Gan J, Zeng D, Li J, Yu H, Zhao H, Yang Y, Tan S, Li G, Luo C, Xie Z, Zhao G, Li H. 2021. Specific microbial taxa and functional capacity contribute to chicken abdominal fat deposition. Front Microbiol 12:643025. doi:10.3389/fmicb.2021.643025. 33815329 PMC8010200

[42] Kim SM, DeFazio JR, Hyoju SK, Sangani K, Keskey R, Krezalek MA, Khodarev NN, Sangwan N, Christley S, Harris KG, Malik A, Zaborin A, Bouziat R, Ranoa DR, Wiegerinck M, Ernest JD, Shakhsheer BA, Fleming ID, Weichselbaum RR, Antonopoulos DA, Gilbert JA, Barreiro LB, Zaborina O, Jabri B, Alverdy JC. 2020. Fecal microbiota transplant rescues mice from human pathogen mediated sepsis by restoring systemic immunity. Nat Commun 11:2354. doi:10.1038/s41467-020-15545-w. 32393794 PMC7214422

[43] Messaoudi S, Manai M, Kergourlay G, Prévost H, Connil N, Chobert JM, Dousset X. 2013. Lactobacillus salivarius: bacteriocin and probiotic activity. Food Microbiol 36:296–304. doi:10.1016/j.fm.2013.05.010. 24010610

[44] Walters WA, Xu Z, Knight R. 2014. Meta-analyses of human gut microbes associated with obesity and IBD. FEBS Lett 588:4223–4233. doi:10.1016/j.febslet.2014.09.039. 25307765 PMC5050012

[45] Yang J, Li Y, Wen Z, Liu W, Meng L, Huang H. 2021. Oscillospira—a candidate for the next-generation probiotics. Gut Microbes 13:1987783. doi:10.1080/19490976.2021.1987783. 34693878 PMC8547878

[46] Martin-Gallausiaux C, Marinelli L, Blottière HM, Larraufie P, Lapaque N. 2021. SCFA: mechanisms and functional importance in the gut. Proc Nutr Soc 80:37–49. doi:10.1017/S0029665120006916. 32238208

[47] Sanna S, van Zuydam NR, Mahajan A, Kurilshikov A, Vich Vila A, Võsa U, Mujagic Z, Masclee AAM, Jonkers DMAE, Oosting M, Joosten LAB, Netea MG, Franke L, Zhernakova A, Fu J, Wijmenga C, McCarthy MI. 2019. Causal relationships among the gut microbiome, short-chain fatty acids and metabolic diseases. Nat Genet 51:600–605. doi:10.1038/s41588-019-0350-x. 30778224 PMC6441384

[48] Zhu L, Sha L, Li K, Wang Z, Wang T, Li Y, Liu P, Dong X, Dong Y, Zhang X, Wang H. 2020. Dietary flaxseed oil rich in omega-3 suppresses severity of type 2 diabetes mellitus via anti-inflammation and modulating gut microbiota in rats. Lipids Health Dis 19:20. doi:10.1186/s12944-019-1167-4. 32028957 PMC7006389

[49] Xu QQ, Ma XW, Dong XY, Tao ZR, Lu LZ, Zou XT. 2020. Effects of parental dietary linoleic acid on growth performance, antioxidant capacity, and lipid metabolism in domestic pigeons (Columba livia). Poult Sci 99:1471–1482. doi:10.1016/j.psj.2019.11.002. 32111316 PMC7587642

[50] Yue H, Qiu B, Jia M, Liu W, Guo X-F, Li N, Xu Z-X, Du F-L, Xu T, Li D. 2021. Effects of α-linolenic acid intake on blood lipid profiles: a systematic review and meta-analysis of randomized controlled trials. Crit Rev Food Sci Nutr 61:2894–2910. doi:10.1080/10408398.2020.1790496. 32643951

[51] Fu Y, Wang Y, Gao H, Li D, Jiang R, Ge L, Tong C, Xu K. 2021. Associations among dietary omega-3 polyunsaturated fatty acids, the gut microbiota, and intestinal immunity. Mediators Inflamm 2021:8879227. doi:10.1155/2021/8879227. 33488295 PMC7801035

[52] Newton PT, Vuppalapati KK, Bouderlique T, Chagin AS. 2015. Pharmacological inhibition of lysosomes activates the MTORC1 signaling pathway in chondrocytes in an autophagy-independent manner. Autophagy 11:1594–1607. doi:10.1080/15548627.2015.1068489. 26259639 PMC4590675

[53] Zhou M, Xu W, Wang J, Yan J, Shi Y, Zhang C, Ge W, Wu J, Du P, Chen Y. 2018. Boosting mTOR-dependent autophagy via upstream TLR4-MyD88-MAPK signalling and downstream NF-κB pathway quenches intestinal inflammation and oxidative stress injury. EBioMedicine 35:345–360. doi:10.1016/j.ebiom.2018.08.035. 30170968 PMC6161481

[54] Xiong W, Wang Y, Sun Y, Ma L, Zeng Q, Jiang X, Li A, Zeng Z, Zhang T. 2018. Antibiotic-mediated changes in the fecal microbiome of broiler chickens define the incidence of antibiotic resistance genes. Microbiome 6:34. doi:10.1186/s40168-018-0419-2. 29439741 PMC5811963

[55] Turcotte C, Thibodeau A, Quessy S, Topp E, Beauchamp G, Fravalo P, Archambault M, Gaucher ML. 2020. Impacts of short-term antibiotic withdrawal and long-term judicious antibiotic use on resistance gene abundance and cecal microbiota composition on commercial broiler chicken farms in Québec. Front Vet Sci 7:547181. doi:10.3389/fvets.2020.547181. 33409294 PMC7779680

[56] Ghanbari M, Klose V, Crispie F, Cotter PD. 2019. The dynamics of the antibiotic resistome in the feces of freshly weaned pigs following therapeutic administration of oxytetracycline. Sci Rep 9:4062. doi:10.1038/s41598-019-40496-8. 30858509 PMC6411716

[57] Gupta CL, Blum SE, Kattusamy K, Daniel T, Druyan S, Shapira R, Krifucks O, Zhu Y-G, Zhou X-Y, Su J-Q, Cytryn E. 2021. Longitudinal study on the effects of growth-promoting and therapeutic antibiotics on the dynamics of chicken cloacal and litter microbiomes and resistomes. Microbiome 9:178. doi:10.1186/s40168-021-01136-4. 34454634 PMC8403378

[58] Maślanka T, Jaroszewski JJ, Mikołajczyk A, Rotkiewicz T. 2009. Effect of increasing doses of enrofloxacin on chicken articular cartilage. Pol J Vet Sci 12:21–33. 19459436

[59] Yin D, Yin X, Wang X, Lei Z, Wang M, Guo Y, Aggrey SE, Nie W, Yuan J. 2018. Supplementation of amylase combined with glucoamylase or protease changes intestinal microbiota diversity and benefits for broilers fed a diet of newly harvested corn. J Anim Sci Biotechnol 9:24. doi:10.1186/s40104-018-0238-0. 29545946 PMC5846306

[60] Fletcher JR, Erwin S, Lanzas C, Theriot CM. 2018. Shifts in the gut metabolome and Clostridium difficile transcriptome throughout colonization and infection in a mouse model. mSphere 3:e00089-18. doi:10.1128/mSphere.00089-18. 29600278 PMC5874438

[61] Edgar RC. 2013. UPARSE: highly accurate OTU sequences from microbial amplicon reads. Nat Methods 10:996–998. doi:10.1038/nmeth.2604. 23955772

[62] Wang Q, Garrity GM, Tiedje JM, Cole JR. 2007. Naive Bayesian classifier for rapid assignment of rRNA sequences into the new bacterial taxonomy. Appl Environ Microbiol 73:5261–5267. doi:10.1128/AEM.00062-07. 17586664 PMC1950982

[63] Edgar RC. 2010. Search and clustering orders of magnitude faster than BLAST. Bioinformatics 26:2460–2461. doi:10.1093/bioinformatics/btq461. 20709691

[64] Schloss PD, Westcott SL, Ryabin T, Hall JR, Hartmann M, Hollister EB, Lesniewski RA, Oakley BB, Parks DH, Robinson CJ, Sahl JW, Stres B, Thallinger GG, Van Horn DJ, Weber CF. 2009. Introducing mothur: open-source, platform-independent, community-supported software for describing and comparing microbial communities. Appl Environ Microbiol 75:7537–7541. doi:10.1128/AEM.01541-09. 19801464 PMC2786419

[65] Caporaso JG, Kuczynski J, Stombaugh J, Bittinger K, Bushman FD, Costello EK, Fierer N, Peña AG, Goodrich JK, Gordon JI, Huttley GA, Kelley ST, Knights D, Koenig JE, Ley RE, Lozupone CA, McDonald D, Muegge BD, Pirrung M, Reeder J, Sevinsky JR, Turnbaugh PJ, Walters WA, Widmann J, Yatsunenko T, Zaneveld J, Knight R. 2010. QIIME allows analysis of high-throughput community sequencing data. Nat Methods 7:335–336. doi:10.1038/nmeth.f.303. 20383131 PMC3156573

[66] Wen B, Mei Z, Zeng C, Liu S. 2017. metaX: a flexible and comprehensive software for processing metabolomics data. BMC Bioinformatics 18:183. doi:10.1186/s12859-017-1579-y. 28327092 PMC5361702

[67] Westerhuis JA, Hoefsloot HC, Smit S, Vis DJ, Smilde AK, van Velzen EJ, van Duijnhoven JP, van Dorsten FA. 2008. Assessment of PLSDA cross validation. Metabolomics 4:81–89. doi:10.1007/s11306-007-0099-6.

[68] Haug K, Cochrane K, Nainala VC, Williams M, Chang J, Jayaseelan KV, O’Donovan C. 2020. MetaboLights: a resource evolving in response to the needs of its scientific community. Nucleic Acids Res 48:D440–D444. doi:10.1093/nar/gkz1019. 31691833 PMC7145518

