# Supplementary material for: Effect of Enrofloxacin on the Microbiome, Metabolome, and Abundance of Antibiotic Resistance Genes in the Chicken Cecum
Source: Microbiol Spectr. 2023 Feb 22;11(2):e04795-22. doi: 10.1128/spectrum.04795-22 (PMC10100749; doi:10.1128/spectrum.04795-22)
Supplement: Supplemental file 1 — Fig. S1 and S2 and Tables S1 to S3. Download spectrum.04795-22-s0001.pdf, PDF file, 0.5 MB [file spectrum.04795-22-s0001.pdf]

SUPPLEMENTARY FIGURES AND FIGURE LEGENDS

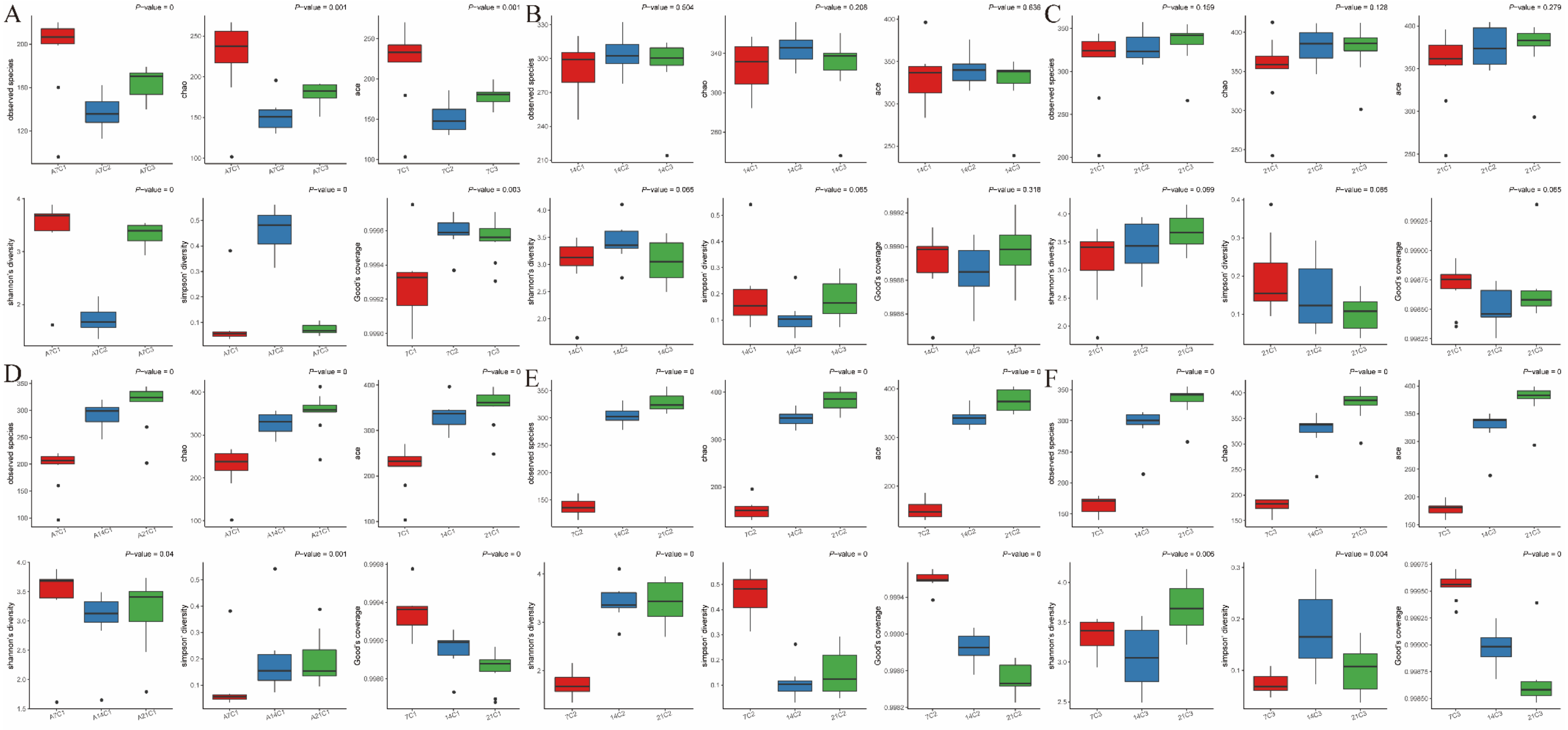

Figure S1. Alpha diversity analysis of the cecal microbiota of chickens after the use of different concentrations of enrofloxacin. (A–C): Alpha diversity of C1, C2, and C3 groups at days 7 (A), 14 (B), and 21 (C). (D–F): Alpha diversity of C1 (D), C2 (E), and C3 (F) groups at the three developmental stages.

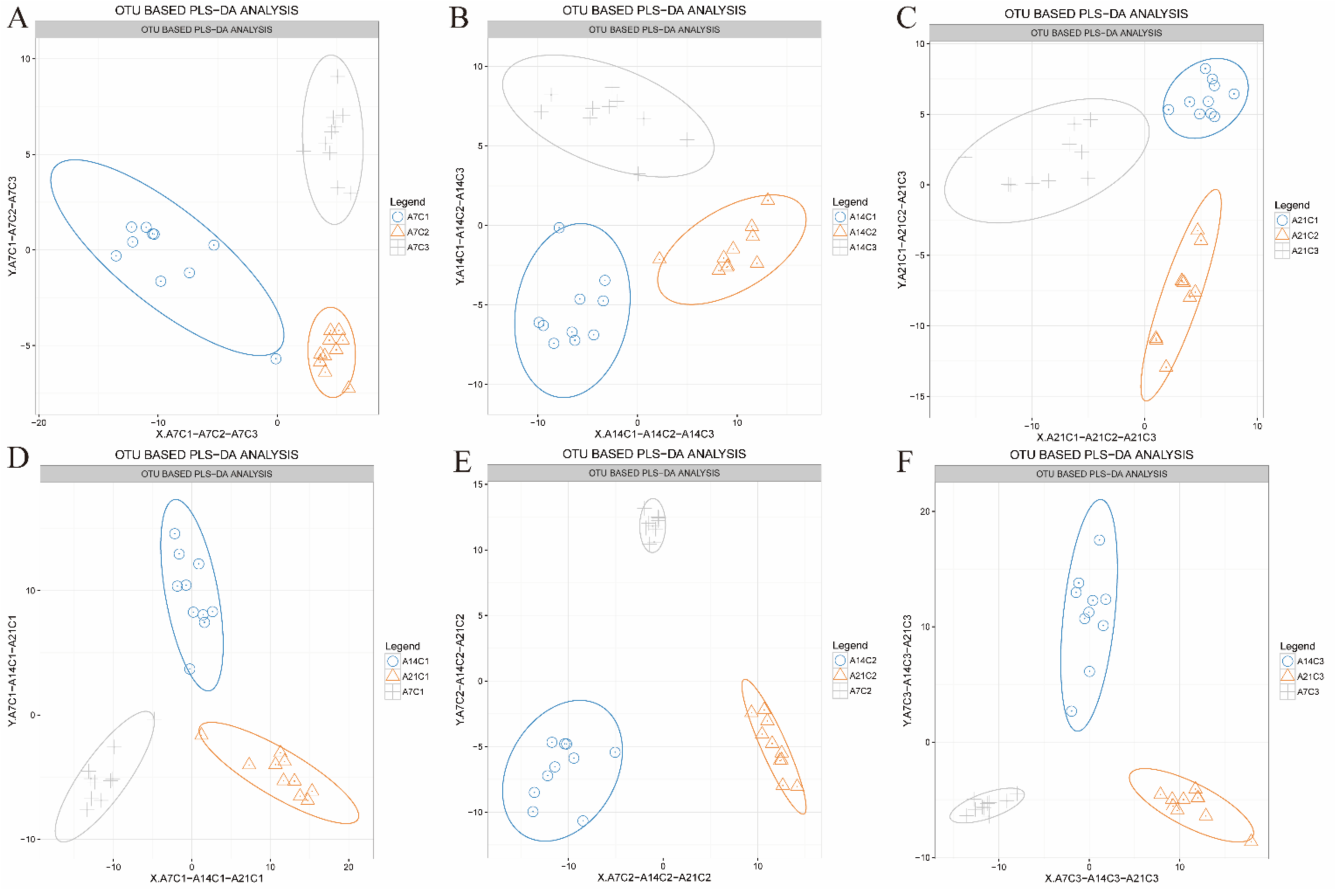

Figure S2. Scatterplot of partial least squares-discriminant analysis (PLS-DA) scores of the cecal microbiota with the first principal component on the horizontal axis and the second principal component on the vertical axis. Numbers in parentheses indicate the score of the principal component and represent the percentage of the total variance explained by the corresponding principal component. (A–C): Composition of the cecal microbiota at days 7 (A), 14 (B), and 21 (C). (D–F): Composition of the cecal microbiota for groups C1 (D), C2 (E), and C3 (F) at the three developmental stages.

SUPPLEMENTARY TABLES

Table S1. Relative abundance of microbial communities in chicken cecum contents at the phylum level

Taxon phylum	Groups								
	A7C1	A7C2	A7C3	A14C1	A14C2	A14C3	A21C1	A21C2	A21C3
Firmicutes	0.814147039	0.306953717	0.941811574	0.538818185	0.773961479	0.568369452	0.550076767	0.651740978	0.710845701
Bacteroidetes	0.103562629	0.673696008	8.61E-05	0.399107514	0.21785234	0.3888286	0.420426237	0.343715847	0.282197658
Proteobacteria	0.082088302	0.019346078	0.058077778	0.061580565	0.007671789	0.042245319	0.027183805	0.001895409	0.002823832
Tenericutes	0.000103479	0	7.38E-06	0.000362073	0.00014207	0.000250249	0.000782926	0.002455057	0.00302971
Actinobacteria	9.12E-05	0	0	0.000115205	0.000320883	0.000294686	0.000213525	0.000192709	0.00044734
Thermi	4.93E-06	0	7.38E-06	0	4.65E-05	0	0	0	0
Cyanobacteria	2.46E-06	2.10E-06	9.84E-06	1.65E-05	4.90E-06	1.17E-05	0.001314197	0	0.000655759
Verrucomicrobia	0	2.10E-06	0	0	0	0	2.54E-06	0	0

Table S2. Relative abundance of microbial communities in chicken cecum contents at the genus level

Taxon	Groups								
Genus	7C1	7C2	7C3	14C1	14C2	14C3	21C1	21C2	21C3
Blautia	0.003471469	0.003032663	0.004054626	0.002680278	0.008075954	0.004417949	0.009908591	0.01421821	0.010957282
Anaerotruncus	0.010776584	0.000220366	2.95E-05	0.003862892	0.000921007	0.017980513	0.004163743	0.003228531	0.005823042
Butyricicoccus	0.013930226	0.006208039	0.018684718	0.005835483	0.010939402	0.007289966	0.004400146	0.004242232	0.004854653
Coprococcus	0.002660885	0.010254387	0.008008439	0.007119195	0.01099819	0.006457362	0.019908693	0.028584251	0.035408464
Dorea	0.011919779	0.002749334	0.008369888	0.012945273	0.024433618	0.013426916	0.01095334	0.010474908	0.010553151
Clostridium	0.030646989	0.024133278	0.045628676	0.014668645	0.020573229	0.014547189	0.011281253	0.009458568	0.012444178
Escherichia	0.074243619	0.009396007	0.057959754	0.061359559	0.007453784	0.0420512	0.026677953	0.001850532	0.002757748
Lactobacillus	0.009938898	0.000791221	0.002726852	0.003731229	0.000987143	0.005175712	0.058150057	0.121079169	0.049077743
Oscillospira	0.182763378	0.045345125	0.15772223	0.075833061	0.02528114	0.046452778	0.058655909	0.064372641	0.054542405
Ruminococcus	0.097735784	0.074078816	0.185767748	0.058763922	0.104539142	0.054297034	0.071403878	0.070109026	0.10323635
Bacteroides	0.103555238	0.673696008	8.61E-05	0.399100461	0.21785234	0.388826262	0.420413527	0.343689449	0.28218495
Other	0.45835715	0.150094758	0.510961504	0.354100003	0.567945053	0.399077119	0.304082909	0.328692484	0.428160036

Table S3. Sequences of Primers Used in RT-qPCR Analysis

Target gene	Primer sequence	Calibration curve	R^2	E
oqxA	F: CTGGATAACTCGCAGCGTCT<br>R: TCGGTCAGTACCGCTTTGTC	y = -3.4367x + 39.957	0.999	95.4%
oqxB	F: CCCTGTACATCCAGGATCGC<br>R: GCCTGGTAAGTCGAGATCGG	y = -3.346x + 40.287	0.994	99.0%
qepA	F: GCCGGTGATGCTGCTGA<br>R: CAGGAACAGCGCCCCGA	y = -3.9427x + 45.485	0.996	99.3%
qnrA	F: ATTTCTCACGCCAGGATTTG<br>R: GCAGATCGGCATAGCTGAAG	y = -3.582 + 38.554	0.999	90.2%
qnrB	F: GGAATAGAAATTCGCCACTG<br>R: TCGCCGTTCGCCAGTCGAA	y = -3.473x + 36.351	0.998	94.1%
qnrD	F: AGTGAGTGTTTAGCTCAAGGAG<br>R: CAGTGCCATTCCAGCGATT	y =- 3.538x + 41.391	0.996	91.7%
qnrS	F: GACGTGCTAACTTGCGTGAT<br>R: TGGCATTGTTGGAAACTTG	y = -3.318x + 38.071	0.992	100.2%
aac (6')-Ib-cr	F: TTGCGATGCTCTATGAGTGGCTA<br>R: CTCGAATGCCTGGCGTGTTT	y =-3.6767x + 39.878	0.998	98.6%
